# The Future of Transforming Healthcare Systems Into Circular Economy Models: Comment on "A Review of the Applicability of Current Green Practices in Healthcare Facilities"

**DOI:** 10.34172/ijhpm.9176

**Published:** 2025-08-09

**Authors:** Edda Weimann

**Affiliations:** ^1^Department of Information Systems, School of IT, University of Cape Town, Cape Town, South Africa.; ^2^Department of Child Health, School of Medicine and Health, Technical University of Munich, Munich, Germany.

**Keywords:** Decarbonising Healthcare, Net-Zero Healthcare, Climate-Change and Health, Decarbonising Supply Chain, Planetary Health

## Abstract

The healthcare sector is both a guardian of health and a significant contributor to global carbon emissions and environmental degradation. In their scoping review, Soares et al explore the applicability of circular economy (CE) principles within healthcare facilities, identifying eight areas for intervention. While their work provides a valuable synthesis, this commentary highlights future points of interest such as vulnerable populations, a call to broaden governance frameworks, and to move from an overly Eurocentric to a more global scope. Low- and middle-income countries (LMICs) face major barriers to implementing CE models in healthcare, including weak policy frameworks, a lack of holistic recycling chains, awareness and training, as well as limited incentives. Stronger government leadership is needed to develop CE policies, foster multi-sector collaboration among private investors, governments, academia, non-governmental organisations, and international partners. Drawing on public health and child health perspectives, and informed by work in net-zero hospital initiatives, this commentary argues for a more transformative, equitable, and globally inclusive vision of circular healthcare. Organisations like Health Care Without Harm (HCWH) can support implementation through technical expertise, advocacy, and capacity-building.

 The world is facing a confluence of existential threats: the escalating climate crisis, severe environmental degradation, and unprecedented biodiversity loss. Paradoxically, the healthcare sector—tasked with protecting public health—is a notable contributor to these crises, accounting for over 5% of global greenhouse gas (GHG) emissions in the post-COVID-19 era.^1,2^ The scoping review by Soares et al,^3^ *A Review of the Applicability of Current Green Practices in Healthcare Facilities*, is therefore a timely and necessary contribution to the growing discourse on environmental sustainability in healthcare systems.

 Soares et al^3^ conduct a comprehensive scoping review using the Arksey and O’Malley framework and the Preferred Reporting Items for Systematic Reviews and Meta-Analyses extension for Scoping Reviews (PRISMA-ScR) checklist,^4^ which enhances transparency and methodological rigour. Their review identifies eight areas where circular economy (CE) principles can be implemented: energy, water, food, transport, hospital design, green procurement, waste management, and behaviour change. These domains align with the World Health Organization’s (WHO’s) sustainability work for healthcare systems^5^ which in turn are based on the United Nations Sustainable Development Goals (SDGs) particularly SDG 3 (Good Health and Well-being) and SDG 13 (Climate Action). As the supply chain contributes 50% to 70% of greenhouse gas (GHG) emissions, environmental degradation and pollution, CE is a powerful and holistic approach to significantly reduce the impacts.

 Particularly commendable is the inclusion of behavioural change and staff engagement as barriers to CE implementation – an often overlooked but crucial element. The integration of CE principles such as green procurement, infrastructure adaptation, and energy efficiency demonstrates a practical understanding of operational levers in healthcare. The authors emphasise that lack of familarity and fear of the unknown may explain why the concept of CE has been slow to take off globally. As a result, CE is still in its infancy. But also the differences in healthcare systems, for example between high- and low-income countries, also play a role, as the source of environmental impacts is distributed differently between local contributions and global supply chains.However, CE plays a central role in focusing healthcare on value by reducing over-diagnosis, over-treatment, and over-prescription—practices that not only strain resources but also contribute significantly to carbon emissions and medical waste. By rethinking how resources are better used across the system, CE fosters a more sustainable and efficient model of care. This transformative potential aligns with the view of the Lancet Commission on Climate and Health, which described the climate crisis as the “greatest global health opportunity of the XXI century.”

 One of the most prominent omissions is the inclusion of vulnerable populations—particularly children, pregnant women, neonates, and the elderly – who are disproportionately affected by environmental stressors.^6,7^ A life-course approach and perspective is essential to ensure that sustainability transitions do not entrench or exacerbate existing health inequalities. Tailored CE interventions for paediatric, maternal and geriatric healthcare settings need to be prioritised for future research and the implementation of solutions.

 While Soares et al identify key CE strategies, there are also systemic drivers. Transforming healthcare systems requires embedding CE principles into leadership, policy mandates, hospital management, and training programmes.^8^ Examples such as the UK’s Greener National Health Service (NHS) initiative highlight the importance of top-down frameworks coupled with staff-level commitment. As Weimann and Weimann^8^ highlight, this systemic shift requires a cultural shift within institutions, where sustainability becomes a shared responsibility integrated into daily clinical and operational decision-making.

 The review largely reflects the European context, influenced by European Union (EU) policy frameworks and the European Green Deal. However, health systems in low- and middle-income countries (LMICs)—often models of resource conservation and innovative reuse—are notably absent from current global approaches. In LMICs, there is a reluctance to adopt CE practices and reduce the use of crude landfills as well as polluting and toxic incinerators because alternatives are rarely available. Limited infrastructure, lack of regulatory support and financial constraints often make it difficult to implement sustainable waste management systems or to invest in reusable medical technologies. Further, vulnerable population groups are experiencing the health impacts of toxic waste, air pollution and microplastic pollution. Therefore, the economic concept of the 3Rs—reduce, reuse, recycle—with the emphasis on “reduce,” offers a valuable approach to minimise health risks while ensuring the safe use of scarce resources. While research on the CE in LMICs is still limited, a study from Asia demonstrates its economic potential, revealing that full recycling can substantially enhance the value of healthcare waste,^9^ aligning with the findings of Weimann and Patel^10^ from South Africa. Best practice examples from the NHS/UK and initiatives such as the Born Green Generation, which aims to reduce plastic exposure within the first 1000 days (https://borngreengeneration.org/), show that significant financial and waste savings can be made when healthcare changes from single-use plastics to reusable products.These insights underscore the critical role of reduction of single plastic devices, source segregation and recycling in reducing environmental and economic burdens and provide a valuable foundation for future research on hazardous waste management in LMIC settings. The restoration and reutilisation of damaged objects within the healthcare sector has the potential to engender employment opportunities in economically disadvantaged settings, as opposed to the perpetual acquisition of new items. In many African countries serious challenges persist in managing healthcare waste.^11^ Core issues include weak infrastructure, absence of national policies, poor waste segregation, and continued reliance on harmful practices such as open burning and poorly maintained incineration. These methods pose considerable health and environmental risks, particularly in resource-limited settings. While some nations, like South Africa and Ghana, have taken steps toward policy development, many still lack effective waste management systems.^11^ Organisations such as Health Care Without Harm (HCWH) can play a key role in supporting implementation by providing technical expertise, advocacy, and capacity-building.

 There is a strong need for coordinated, environmentally sustainable, and context-specific solutions. These local contexts offer important insights for developing resilient and equitable CE models, especially in areas with severe resource constraints.^12^ A truly global CE framework must be co-produced with, and informed by, the experiences of LMICs,^10^ not only to ensure its relevance and feasibility across diverse healthcare systems, but also to address historical inequities and promote inclusive, context-sensitive solutions that leave no region behind.

 The concept of net-zero hospitals is typically framed around emissions accounting and infrastructural change. However, to be transformative, such institutions must also embody environmental ethics in healthcare delivery, procurement, and education. As highlighted by Sherman et al^13^ and Rizan et al,^14^ sustainability is not just about metrics—it is about systemic redesign and cultural change.

 The scoping review identifies gaps and opportunities for future research. Empirical data on carbon footprints and GHG emissions of national healthcare systems are clearly lacking. This makes it difficult to demonstrate the effectiveness of CE interventions in reducing carbon emissions. Future studies should include longitudinal data to measure outcomes across CE domains.^15^ Despite the CE importance in the EU, only 6 out of 27 EU countries have conducted climate change and vulnerability assessments.

 Novel technologies such as artificial intelligence (AI)-driven energy management, biodegradable medical materials, and blockchain-enhanced sustainable procurement exhibit promise yet remain underexplored. In contrast, telemedicine and digital health, by reducing the need for travel and cutting resource use, offer a more immediate and significant impact on decarbonising healthcare—a value that has grown even more evident following the digital acceleration spurred by the pandemic. A deeper comparative research focus of CE policy implementation in different healthcare systems including LMICs would provide transferable lessons.^8,16^ More attention is needed to promote safer alternatives to hazardous incineration and crude landfilling in medical waste management. Sustainable technologies like autoclaving, anaerobic digestion, plasma gasification, and the use of eco-friendly material substitutes remain significantly underused.^9,17^ CE offers solutions, but holistic CE models need to be in place before they can be successfully rolled out. Implementing CE and meeting an ambitious low carbon economic target would allow for a 4% increase in the workforce as claimed by Soares et al.^3^

 Soares et al^3^ offer a foundational synthesis of CE applications in healthcare, but the path forward requires a shift from technical optimisation to holistic transformation. CE needs to be reimagined as a regenerative ethos, centred on equity, resource sustainability and goverance reform. Only then can healthcare systems fulfil their dual mandate: to heal people and to heal the planet.

## Ethical issues

 Not applicable.

## Conflicts of interest

 Author declares that she has no conflicts of interest.

## References

[1] Arup. Healthcare’s carbon footprint. Health Care’s Climate Footprint. 2019. https://www.arup.com/globalassets/downloads/insights/healthcares-carbon-footprint.pdf.

[2] Eckelman MJ, Sherman J (2016). Environmental impacts of the US health care system and effects on public health. PLoS One.

[3] Soares AL, Buttigieg SC, Bak B (2023). A review of the applicability of current green practices in healthcare facilities. Int J Health Policy Manag.

[4] Tricco AC, Lillie E, Zarin W (2018). PRISMA extension for scoping reviews (PRISMA-ScR): checklist and explanation. Ann Intern Med.

[5] World Health Organization (WHO). Environmentally Sustainable Health Systems: A Strategic Document. WHO Regional Office for Europe; 2017.

[6] Ebi KL, Balbus J, Luber G, et al. Human health. In: Impacts, Risks, and Adaptation in the United States: Fourth National Climate Assessment, Volume II. Washington, DC: U.S. Global Change Research Program; 2018.

[7] Etzel RA, Weimann E, Homer C (2024). Climate change impacts on health across the life course. J Glob Health.

[8] Weimann L, Weimann E (2022). On the road to net zero health care systems: governance for sustainable health care in the United Kingdom and Germany. Int J Environ Res Public Health.

[9] Ali M, Geng Y (2018). Accounting embodied economic potential of healthcare waste recycling-a case study from Pakistan. Environ Monit Assess.

[10] Weimann E, Patel B (2016). Tackling the climate targets set by the Paris agreement (COP 21): green leadership empowers public hospitals to overcome obstacles and challenges in a resource-constrained environment. S Afr Med J.

[11] Chisholm JM, Zamani R, Negm AM (2021). Sustainable waste management of medical waste in African developing countries: a narrative review. Waste Manag Res.

[12] Health Care Without Harm. Global Road Map for Health Care Decarbonization. 2019. https://noharm-global.org/documents/global-road-map-health-care-decarbonization.

[13] Sherman JD, Thiel C, MacNeill A (2020). The green print: advancement of environmental sustainability in healthcare. Resour Conserv Recycl.

[14] Rizan C, Steinbach I, Nicholson R, Lillywhite R, Reed M, Bhutta MF (2020). The carbon footprint of surgical operations: a systematic review. Ann Surg.

[15] Malik A, Lenzen M, McAlister S, McGain F (2018). The carbon footprint of Australian health care. Lancet Planet Health.

[16] Pichler PP, Jaccard IS, Weisz U, Weisz H (2019). International comparison of health care carbon footprints. Environ Rese Lett.

[17] Ali M, Wang W, Chaudhry N, Geng Y (2017). Hospital waste management in developing countries: a mini review. Waste Manag Res.

